# Titanium mesh for bone augmentation in oral implantology: current application and progress

**DOI:** 10.1038/s41368-020-00107-z

**Published:** 2020-12-30

**Authors:** Yu Xie, Songhang Li, Tianxu Zhang, Chao Wang, Xiaoxiao Cai

**Affiliations:** 1 State Key Laboratory of Oral Diseases & National Clinical Research Center for Oral Diseases & Dept. of Head and Department of Implant Dentistry, West China Hospital of Stomatology, Sichuan University, Chengdu, China; 2 College of Stomatology, Chongqing Medical University, Chongqing, China; 3 Beijing Advanced Innovation Center for Biomedical Engineering, Beihang University, Beijing, China

**Keywords:** Outcomes research, Oral diseases

## Abstract

Guided bone regeneration (GBR) is an effective and simple method for bone augmentation, which is often used to reconstruct the alveolar ridge when the bone defect occurs in the implant area. Titanium mesh has expanded the indications of GBR technology due to its excellent mechanical properties and biocompatibility, so that the GBR technology can be used to repair alveolar ridges with larger bone defects, and can obtain excellent and stable bone augmentation results. Currently, GBR with titanium mesh has various clinical applications, including different clinical procedures. Bone graft materials, titanium mesh covering methods, and titanium mesh fixing methods are also optional. Moreover, the research of GBR with titanium mesh has led to multifarious progresses in digitalization and material modification. This article reviews the properties of titanium mesh and the difference of titanium mesh with other barrier membranes; the current clinical application of titanium mesh in bone augmentation; common complications and management and prevention methods in the application of titanium mesh; and research progress of titanium mesh in digitization and material modification. Hoping to provide a reference for further improvement of titanium mesh in clinical application and related research of titanium mesh.

## Introduction

In oral implantology, the quality and volume of alveolar bone in the implant area affect implant position, primary stability and soft tissue shape recovery, and other critical factors related to satisfactory implantation restoration.^1^ Generally, the alveolar bone will suffer secondary absorption and atrophy after tooth loss, the width and height of the alveolar ridge will decrease, and become insufficient for implantation over time.^2^ Therefore, the reconstruction of alveolar bone in the implant area is a key point in oral implantology. There are many clinical methods for alveolar bone defect recovery, including guided bone regeneration technique (GBR), onlay bone grafting, bone extrusion technique, bone splitting technique, and distraction osteogenesis. Due to its simple operation, low technical sensitivity, osteogenic stability, and multidirectional osteogenesis ability, GBR is one of the most currently used technique to repair alveolar bone defects.^3^

The theory of GBR technology is to selectively prevent epithelial cells and connective tissue cells from bone defect area through barrier membrane based on different migration rate of various cells, allowing osteoblasts preferentially enter the bone defect area to complete bone induction and regeneration. Meanwhile, bone graft materials are placed in the bone defect area as scaffolds, and guiding osteoblasts and osteocyte to form new bone.^4^ Previous studies have shown that, in the clinical application of GBR, the bone defect area’s spatial support may play a more critical role than the cell-selective isolation.^5^ If the bone grafts in the defect area lack support, it may be forced to shift by local stress, resulting in the collapse of bone-augmented area, which cannot achieve the expected effect. Therefore, for the barrier membranes in GBR technology, on the premise of good biocompatibility, it is ideal to have sufficient stiffness, supportability, and retention capacity. However, although traditional barrier membranes (such as absorbable collagen membrane, nonabsorbable expanded polytetrafluorethylene membrane (ePTFE), etc.) have the property of cell-selective isolation, they are relatively soft and difficult to provide adequate retention and protection for the bone regeneration areas.^6^ Hence, when the traditional barrier membranes be applied to large bone defects, limited by its stiffness, it is difficult to maintain a suitable and stable bone regeneration space, and is easy to generate micromotion that affects blood supply.^7^ When the alveolar bone has severe vertical or horizontal bone defects, many clinical studies suggest that titanium mesh shows superior mechanical properties and great osteogenic performance during application.^8–10^ Therefore, this review intends to discuss the application and progress of titanium mesh in GBR.

This review summarized the properties of titanium mesh, differences of titanium mesh with other barrier membranes, current clinical application of titanium mesh in bone augmentation, common complications of titanium mesh and its management or prevention, and the research progress of titanium mesh. In order to provide a reference for the improvement of titanium mesh in research and clinical application.

## Properties of titanium mesh

Titanium is widely used in surgical operations due to its high stiffness, low density, corrosion resistance, and good biocompatibility. Titanium mesh, as its application product, has unique characteristics as a GBR barrier membrane for bone augmentation.

### Mechanical properties

Titanium mesh has good mechanical properties, its high strength and stiffness enable space support for osteogenesis, its stability is necessary to maintain bone graft volume during wound healing, and the elasticity can reduce the oppression of oral mucosa.^11^ Due to its good plasticity, titanium mesh can adapt to various bone defects through bending and shaping. These features enable GBR with titanium mesh to show a high stable osteogenesis effect, and achieve coinstantaneous bone augmentation in horizontal and vertical directions.^5^

For different titanium mesh, the thickness and porosity are the key factors affecting its mechanical properties. Study suggested that the thickness of titanium mesh may affect the total amount of new bone formation, while the pore size may affect the proportion of bone tissue and soft tissue formation under titanium mesh.^12^ The thickness of titanium mesh is directly proportional to its mechanical properties, which commonly used ranges from 0.1 to 0.6 mm currently. Usually, the titanium mesh at 0.2 mm can be suitable for most instances.^13^ Under this thickness, titanium mesh can provide sufficient stiffness to maintain space and protect grafts, while offer appropriate flexibility that reduces the risk of tissue rupture. With the increase of reconstruction area of alveolar ridge, thicker titanium mesh should be used in GBR to maintain bone regeneration space. As for thick titanium mesh, there are often some sharp edges in the process of bending titanium mesh due to the decrease of plasticity, which is closely related to the mucosal rupture titanium mesh exposure, many researches focus on finding a more suitable thickness of titanium mesh. A study showed that 100–200 μm is the ideal thickness of titanium mesh to reconstruct a large number of bone defects.^14^ Consistent with the result, Rakhmatia et al. compared the bone augmentation effect with titanium mesh at 20, 50, and 100 μm in mouse model and concluded that compared with thinner Ti-mesh, the use of titanium mesh at 100 μm can achieve more extensive bone regeneration effect.^12^ Owing to the thinner titanium mesh is rarely used in clinic currently, when it comes to thinner titanium mesh’s clinical application, a balance between the strength for spatial stability and malleability for adapting adjacent bone contours must be found.

In terms of pore size, it also affects the performance of titanium mesh during bone augmentation. The pore of the titanium mesh is thought to play an essential role in establishing blood supply and facilitating metabolic processes of the grafts at the defect site.^15^ Celletti et al. demonstrated that without pores on titanium mesh, exposure of the mesh would occur in 3 weeks after surgery.^16^ However, the relationship between the pore size of titanium mesh and bone formation is still controversial. For the existence of pores on titanium mesh, it is difficult to achieve selective cell isolation, and soft tissue often grow under titanium mesh. Therefore, many studies have attempt to investigate the relationship between the pore size and the amount of soft tissue growth. A study suggested that compared to the titanium mesh with small diameter (0.6 mm), the titanium mesh with large diameter (1.2 mm) promoted more bone regeneration and prevented soft tissue growth more effectively.^15^ This phenomenon may be related to the increased distribution of blood supply, and diffusion of nutrients and oxygen leaded by the large aperture. On the contrary, a study showed that the use of titanium mesh with large diameter (>2 mm) may lead to more soft tissue growth upon the surface of new bone than the use of titanium mesh with small diameter.^17^ A similar result for GBR with polyester meshes in different pore size may explain this contradictory phenomenon: the larger the pore size of the barrier membrane, the more connective tissue grows between the membrane and the regenerated bone, and the more rapid bone regenerate, while the pore size does not make much difference to the amount of bone formation after the osteogenesis stabilized.^3^

### Biological properties and osteogenic property

Titanium mesh has good biocompatibility and can be compatible with tissues. The biocompatibility of materials can be divided into corrosion resistance and cytotoxicity. Due to its low electrical conductivity, titanium is prone to perform electrochemical oxidation to form a passive and inert oxide layer.^18^ This oxide layer can be retained under the pH of human body, leading to high and persistent corrosion resistance in titanium.^19^ Hence, little amount of metal particles can be released from titanium mesh, while the titanium particles has no significant effect on human cells’ relative growth rate.^20^ Study observed that after alveolar ridge reconstruction conducted by titanium mesh, a thin layer of 1–2 mm thick, soft tissue can often be found upon the regenerated bone surface, called “pseudo-periosteum”.^21^ The formation of this soft tissue layer may be related to the insufficient cell exclusion ability of titanium due to its pores. The role of pseudo-periosteum may be related to bone graft protection, graft infection prevention, and absorption. Currently, it is often be removed with titanium mesh in subsequent operation.^22^

According to the currently reported literature, GBR with titanium mesh has strong osteogenesis predictability, and both horizontal and vertical bone augmentation can be obtained in the process with delayed or simultaneous implantation. In the delayed implantation strategy of bone augmentation, most researchers have gained an average bone augmentation of 4–5 mm in bone width and 5–7 mm in bone height.^10,23–26^ While in the strategy of simultaneous implantation with bone augmentation, although there are few researches about three-dimensional bone increment, the realization of ~3–4 mm average bone gain in width and height seems feasible.^27,28^ However, a meta-analysis about the horizontal or vertical bone augmentation effect of titanium mesh could not be performed for the heterogeneity of the data.^13^

Compared to other methods, bone resorption due to infection is rare in the application of titanium mesh.^29–31^ As a small amount of peri-implant bone resorption usually occurs after implant loading,^32,33^ GBR with titanium mesh will also experience small amount of bone resorption. Zhang et al. showed that for a single anterior tooth defect, during the 41-month follow-up period after implant placement, the labial bone plate experienced an average of −0.81 ± 1.00 mm vertical absorption, which bone upon the implant absorbed at mean of 0.13 ± 1.19 mm in horizontal dimension.^28^ And the study conducted by Poli et al. showed that for a large range of GBR with titanium, the mesial and distal bone resorption were at an average of 1.743 ± 0.567 and 1.913 ± 0.71 mm, respectively, during the 88-month follow-up period after implantation.^34^ Therefore, the possibility of bone resorption also needed to be considered when performing bone augmentation with titanium mesh.

### Differences with other barrier membranes

Nowadays, the barrier membranes commonly used in clinical can be divided into absorbable and nonabsorbable membranes according to their absorbability. The main absorbable membrane is collagen membrane, and the main nonabsorbable membranes are ePTFE, titanium-reinforced PTFE, and titanium mesh.^35^ Among the barrier membranes mentioned above, titanium mesh is the only one entirely made of metal. It exploit the advantages of titanium in mechanical and biological properties to the full, performing excellent in space maintenance and bone reconstruction.^13^ In comparison, though some enhanced absorbable collagen membranes can provide spatial protection to the bone graft material at the initial placement, they will gradually degrade with the absorption of the membrane, making them unable to achieve the same spatial maintenance ability as titanium mesh.^7^ Konstantinidis et al. compared the effect of collagen membrane and titanium mesh in the vertical bone augmentation, and found that the collagen membrane group gained 2.77 ± 1.97 mm bone height, and the titanium mesh group gained 4.56 ± 1.74 mm bone height (*p* < 0.05),^36^ which can be considered that titanium mesh has certain advantages in bone augmentation effect. Although Cucchi et al. proved that there is no significant difference (*p* < 0.05) between titanium mesh and titanium-reinforced PTFE in vertical bone augmentation and complication rates.^27^ Researches showed that different from absorbable membranes or other nonabsorbable barrier membranes like ePTFE, it is rare to observe consequent infection of bone regeneration failure in bone-augmented sites after exposure of titanium mesh.^37,38^ This may be related to the dense and surface structure of titanium that is less susceptible to be adhered by bacteria, and the protective effect of pseudo-periosteum formed under titanium mesh.^37,39^ Due to the presences of pores, titanium mesh may lead to spontaneously heal of mucosa upon bone reconstruction area after exposure, which means unlike ePTFE or titanium-reinforced PTFE, it may not need to remove titanium mesh immediately as there is no infection after mesh exposure.^40^

However, titanium mesh also has shortcomings. Unlike absorbable membranes, titanium mesh cannot be resorbed by the body, which means the titanium mesh and fixation screws need to be removal through second-stage surgery, causing trauma to the patients. Besides, different form other absorbable and nonabsorbable barrier membranes, due to its stiffness, titanium mesh needs to be shaped during surgery to adapt profile of alveolar ridge. This process is technically sensitive, time-consuming, and laborious. And the sharp edges will inevitably form during bending, which may stimulate the mucosa, leading to mucosal rupture and exposure of titanium mesh.^41^

## Current application of titanium mesh in bone augmentation

### Indications of titanium mesh

GBR technique with titanium mesh was initially used to fix bone defects for maxillofacial bone reconstruction. Gradually, it is applied to bone reconstruction on alveolar bone defects. Since 1996, Von Arx et al. used titanium mesh to stabilize autologous bone grafts for local alveolar ridge reconstruction to meet the initial stability and osseointegration requirements of implantation.^42^ In this series of cases, titanium mesh showed outstanding biocompatibility and mechanical properties, proving itself is an effective GBR barrier membrane to repair bone defects. Due to its superior mechanical strength and biocompatibility, titanium mesh can achieve a desirable effect in horizontal, vertical, and three-dimensional bone defects in bone augmentation surgery.

Currently, there are various clinical procedures for bone augmentation with titanium mesh, which can be roughly divided into titanium mesh bone augmentation in simultaneous implantation, titanium mesh bone augmentation with delayed implantation, and GBR with titanium mesh in combination with other bone augmentation methods. The relevant clinical studies using GBR with titanium mesh for alveolar ridge reconstruction in the past 10 years are summarized in Table 1. For selecting different bone augmentation procedures with titanium mesh, under the premise of the patient’s general condition assessment, the operative area’s alveolar bone defect should be evaluated in detail. Based on the Terheyden classification, the alveolar ridge defects after tooth extraction can be divided into four types according to the relationship between the bone defect and the expected implantation site.^43^ The specific classification is as follows:

**Table 1:** Summary of clinical studies using titanium mesh for GBR

References	Titanium mesh	No. of patients	Augmented direction	Graft materials and covering	Fix method	Complication	Bone augmentation outcome	Implant placement
Torres^65^	Commercial	15: Control (mesh only); 15: Study (mesh + PRP)	Combination	Xenograft bone; Covering PRP in study group	Micro-screws	Exposure: control: 28.5%, study: 0%; Augmentation failure: control: 4.8%, study: 0%	ABH: control: (3.1 ± 0.8) mm, study: (3.5 ± 0.7) mm; ABW: control: (3.7 ± 0.6) mm, study: (4.1 ± 0.6) mm	Delayed: 6 months
Misch^61^	Commercial	5	Unclear	rhBMP/ACS and bone allograft (20% by volume); No covering	Monocortical screws	No	Success rate: 100%	Delayed: 6 months
Her^17^	Commercial	26	Horizontal/combination	Xenograft bone or autogenous bone; No covering	Micro-screws	Exposure: 26%; Infection: 3.7%	Success rate: 100%	Delayed: 5.7 months
Miyamoto^23^	Commercial	41	Combination	Autogenous bone; No covering	Small screws	Exposure: 36%; Partial bone resorption: 10%; Augmentation failure: 8%	Success rate: 88%; ABH: (8.1 ± 4.8) mm; ABW: (4.3 ± 2.0) mm	Delayed
De Freitas^62^	Commercial	12: Control (autogenous bone); 12: Study (rhBMP-2/ACS)	Horizontal	rhBMP-2/ACS or Autogenous bone; No covering	Screws	No	ABW: control: (3.7 ± 1.4) mm, Test: (3.2 ± 0.9) mm	Delayed: 6 months
Lizio^22^	Custom	12	Combination	Autogenous bone/Xenograft bone 70/30; No covering	Micro-screws	Early exposure: 46.6%; Late exposure: 33.3%	RBV: 1.04 cm^3^ (range 0.37–2.58 cm^3^)	Delayed: 8.6 months
Poli^34^	Commercial	13	Combination	Autogenous bone/xenograft bone 50/50; No covering	Cortical screws	Exposure: 7.69%	Success rate: 100%	Delayed: 6 months
Jung^11^	Custom	10	Horizontal	Autogenous bone/xenograft bone 50/50; No covering	Implant and accessories	Exposure: 30%	Success rate: 100%	Immediate
Konstantinidis^36^	Commercial	26: Collagen membrane or Ti-mesh	Horizontal	CPS and bone allograft; No covering	Unclear	Exposure: collagen membrane: 7.41%, Ti-mesh: 33.33%	ABW: collagen membrane: (2.77 ± 1.97) mm, Ti-mesh: (4.56 ± 1.74) mm	Immediate
Sumida^77^	Custom and commercial	13: Custom; 13: Commercial	Unclear	Autogenous bone; No covering	Screws	Mucosal rupture: custom: 7.7%, commercial: 23.1%; Infection: custom: 7.7%, commercial: 23.1%	Success rate: custom: 92.3%, commercial: 76.9%	Custom: immediate; Commercial: immediate or delayed
Misch^91^	Commercial	15	Vertical	rhBMP-2/ACS and bone allograft (50% by volume); Covering non cross-linked collagen membrane and/or PRP in some cases	Screws	No	ABH: (8.53 ± 3.5) mm	Delayed: 6 months
Ribeiro^26^	Commercial	5	Horizontal	rhBMP/ACS; No covering	Screws	No	Success rate: 100%; ABW: (3.8 ± 0.7) mm	Delayed: 7 months
Uehara^8^	Commercial	21	Horizontal/vertical/combination	Autogenous bone ± Hydroxyapatite; No covering	Micro-screws	Exposure: 60%; Inflammation or infection: 44%	Success rate: 56.6%	Delayed: 5 months
Zita Gomes^47^	Commercial	25	Horizontal	Xenograft bone; Covering absorbable collagen membrane in some cases (*n* = 12)	Implant with accessories	Exposure: 24%; Partial graft loss: 4%; Complete graft loss: 4%	Success rate: 97.5%; ABW: (3.67 ± 0.89) mm	Immediate
Cucchi^27^	Commercial	20: Group A (d-PTFE titanium-reinforced membranes); 19: Group B (Ti-mesh)	Combination	Autogenous bone/bone allograft 50/50; Covering cross-linked collagen membranes in group B	Miniscrews	Surgical complications: group A: 5%, group B: 15.8%; Major healing complications: group A: 10%, group B: 15.8%; Minor healing complications: group A: 5%, group B: 5.3%	Success rate: group A: 95%, group B: 89.5%; VBG: group A: (4.2 ± 1.0) mm, group B: (4.1 ± 1.0) mm; IBD/FBD: group A: (3.8 ± 0.7/−0.5 ± 0.6 mm, group B: 4.0 ± 0.8/−0.2 ± 0.7 mm	Immediate
Sagheb^10^	Custom	17	Combination	Autogenous bone ± xenograft bone; Covering absorbable collagen membrane or absorbable collagen membrane + PRF membranes	Screws	Exposure: 33%	Success rate: 100%; ABH: (6.5 ± 1.7) mm; ABW: (5.5 ± 1.9) mm	Delayed: 6 months
Ciocca^24^	Custom	9	Vertical	Autogenous bone/xenograft bone 50/50; No covering	Osteosynthesis screws	Early exposure: 33%; Late exposure: 33%	ABH: (3.89 ± 1.46) mm	Delayed: 6–8 months
Mounir^9^	Custom	8: Control (custom Ti-mesh), 8: Study (custom peek mesh)	Combination	Autogenous bone/xenograft bone 50/50; Covering collagen membranes	Micro-screws	Early exposure: control: 12.5%, study: 12.5%	Success rate: both 100%; RBV: control: 20.9% ± 13.3%, study: 31.8% ± 22.7%	Delayed: 6 months
Zhang^28^	Custom	12	Combination	Xenograft bone; Covering absorbable collagen membrane	Absorbable sutures	Early exposure: 6.25%	ABH: (3.61 ± 1.50) mm; ABW: (3.10 ± 2.06) mm	Immediate
Ghanaati^72^	Custom	7	Combination	Solid sticky BSM granule-solid PRF and i-PRF mixture; Opening heal, covering PRF-collagen matrix, A-PRF, and PTEF-based membrane or sterile latex	Screws	Exposure during the whole healing stage	Success rate: 100%	Delayed: 4–8 months
Hartmann^68^	Custom	55	Combination	Autogenous bone/xenograft bone 50/50; Covering A-PRF in some cases (*n* = 12)	Osteosynthesis screws	Exposure: 25%; Patial graft loss: 11.8%; Complete graft loss: 1.5%	Success rate: 100%	Immediate or delayed: 4–8 months;
Maiorana^92^	Custom	5	Horizontal	Autogenous bone/xenograft bone 50/50; No covering	Implant with cover screw	Delayed exposure: 25%; Cover screw loss: 12.5%; Edema: 12.5%	ABW: clinically: (4.95 ± 0.96) mm, radiographically: (5.06 ± 0.88) mm	Immediate

rhBMP/ACS, recombinant human bone morphogenetic proteins/absorbable collagen sponge; CPS, calcium phosphosilicate; PRP, platelet-rich plasma; PRF, platelet-rich fibrin; ABH, average bone height gained; ABW, average bone width gained; RBV, reconstructed bone volume; VBG, vertical bone gain; IBD, initial peri-implant bone defect; FBD, final peri-implant bone defect

- (a) Type 1/4: in the initial stage of bone resorption, the reduction of the buccal bone is <50% of the expected implant length, which is usually presented in single tooth loss;
- (b) Type 2/4: the buccal bone wall absorbed to form a blade-like alveolar ridge, the height of alveolar ridge is not reduced while the buccal bone wall absorbed >50% of the expected implant length;
- (c) Type 3/4: after several years of tooth loss, bone resorption resulted in a partial reduction in the height of alveolar ridge, as well as a reduction in the width;
- (d) Type 4/4: after several years of tooth loss, bone resorption achieved full alveolar ridge reduction on height and width.

**Fig. 1:**
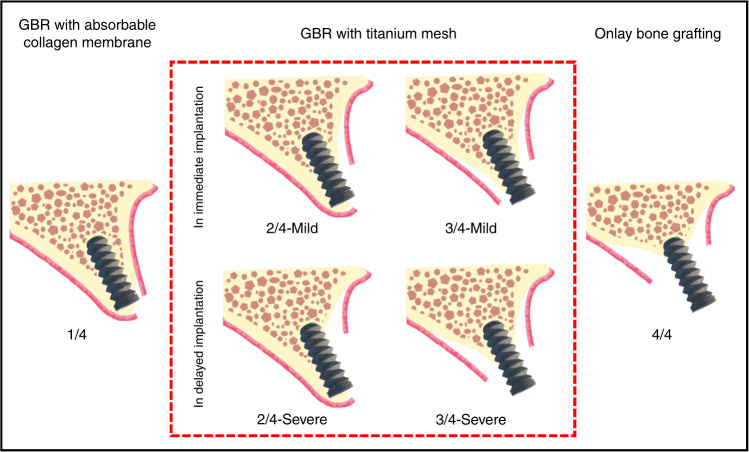
Bone augmentation methods with different types of bone defect. The four types of 1/4, 2/4, 3/4, and 4/4, were classified according to the buccal and palatal relationship between the expected implant placement and the bone defect; the two subtypes of mild and severe were classified according to the severity of vertical absorption in the buccal and palatal wall

For types with slight bone defects (type 1/4), traditional GBR technology can be selected for bone augmentation.^44^ For types with massive bone defects (type 4/4), Terheyden et al. suggested that the bone augmentation method based on onlay bone grafting should be adopted to meet the needs of severely atrophic alveolar ridge reconstruction.^45^ And for types with moderate bone defect (type 2/4 and 3/4), GBR with titanium mesh can be conducted for alveolar ridge reconstruction. And specific procedures of simultaneous or delayed implantation need to be further subdivided on the basis of these two bone defect types. According to the theory of Li et al., types 2/4 and 3/4 can be subclassified into mild and severe based on the degree of vertical absorption on the buccal or palatal bone wall.^46^ Utilizing the excellent bone augmentation and maintenance ability, for alveolar ridges with light buccal or palatal plate absorption in vertical direction (mild type 2/4 or 3/4), GBR with titanium mesh can be applied in simultaneous implantation, as the implant can obtain sufficient initial stability. For alveolar ridges with severe vertical resorption on the buccal or palatal plate (severe type 2/4 or 3/4), excessive threads would be exposed with simultaneous implantation procedure, which may lead to failure of implantation owing to subsequent micromotion of the implant. Hence, it is more recommended to conduct titanium mesh bone augmentation with delayed implantation.^46^ Figure 1 shows the suitable bone augmentation methods for different types and subtypes of alveolar ridge defects. However, the selection of these procedures still needs to be analyzed according to the clinical situation.

### Application of titanium mesh in simultaneous implantation

Nowadays, more and more studies began to utilize the titanium mesh’s excellent ability in maintaining stable osteogenesis space. Researchers attempt to apply titanium mesh in bone augmentation with simultaneous implantation to reduce the trauma and osteogenesis interference in secondary implant surgery, and shorten the whole treatment process. Due to the titanium mesh’s excellent mechanical and biological properties, the augmented alveolar ridge usually does not undergo apparent absorption or deformation.^47^ The results of animal experiments conducted by Artzi et al. showed no statistically significant differences between titanium mesh bone augmentation with staged implantation compared to the results of titanium mesh bone augmentation in simultaneous implantation, and both of which led to similar and persistent osseointegration.^48^ In 1999, Von Arx et al. conducted a clinical study using particle autogenous bone with titanium mesh to restore the horizontal bone defects around implants that placed simultaneously. The postoperative healing was generally good, and after 6 months that the titanium mesh was taken out, the average bone filling rate was 93.5% (ref..^49^) Although it demonstrated that the GBR technique with titanium mesh in immediate implantation is an effective bone augmentation method, it still lacked follow-up data to assess implant survival rate. Later, to treat patients with bone dehiscence or fenestration during implant placement, Jung et al. used the mixture of allograft and autologous bone graft, and preformed titanium mesh in immediate implantation.^11^ This study achieved relatively successful bone regeneration, and 1-year follow-up showed that all implants were successfully regained chewing function. Konstantinidis et al. obtained an average (4.56 ± 1.74) mm of horizontal bone augmentation by GBR with titanium mesh in immediate implantation, although the titanium mesh exposure rate was as high as 33.3%, the implant was not exposed, and the bone-augmented results were not significantly affected.^36^ Although some achievements have been made in various studies on GBR with titanium mesh in immediate implantation, most studies only reported unidirectional bone augmentation in horizontal or vertical, while placing implant simultaneously. Cucchi et al. placed implants in the alveolar ridge and simultaneously used titanium mesh to restore bone defects. After that, not only did the implants obtain sufficient initial stability, but also the peri-implant bone defect ((4.0 ± 0.8) mm before bone augmentation) was repaired ((−0.2 ± 0.7) mm after bone augmentation) through simultaneous bone augmentation, and the vertical bone was gained at the average of (4.1 ± 1.0) mm (ref..^27^) In 2019, Zhang et al. used L-shaped titanium mesh to conduct GBR at the same time of implantation. In the patients with a small or medium alveolar bone defect requiring both horizontally and vertically bone augmentation, the mean horizontal and vertical bone augmentation reached (3.10 ± 2.06) and (3.61 ± 1.50) mm, which gained enough three-dimensional bone volume, and achieved a 93.75% success rate of implantation.^28^ A systematic review that analyzed simultaneous implantation after bone augmentation with titanium mesh demonstrated the survival rate of implants placed into native bone and that placed immediately after bone grafting is similar, and in the statistics of this article, no implant failure is observed.^37^ Therefore, it can be concluded that under the premise of indications, the immediate implantation after bone augmentation with titanium mesh is a feasible and favorable treatment strategy.

### Application of titanium mesh with delayed implantation

From the time titanium mesh was used as a barrier membrane for GBR, in most cases, the bone augmentation with titanium mesh was carried out in stages with implant placement. After the bone reconstruction at alveolar bone defect becomes stable, implants were placed in an appropriate position during secondary surgery. This method has certain guarantees for the osteogenic consequence, and even if the first bone augmentation surgery fails, the alveolar ridge reconstruction can still be performed at subsequent implant surgery without affecting the effects of implantation. Hence, many studies attempted to apply GBR with titanium mesh to atrophied edentulous alveolar ridge for bone augmentation. Poli et al. performed titanium mesh augmentation on patients with edentulous jaws, achieved ideal bone regeneration after a 6 months recovery period, and allowed the implant to be implanted in the ideal position. During the 88-month follow-up, the new bone obtained by the titanium mesh augmentation had very little resorption and did not affect the function of the implant.^34^ Ciocca et al. also used GBR with titanium mesh to reconstruct extensive alveolar ridge defects, and a mean vertical bone of (3.89 ± 1.46) mm were gained after recovery period of 6–8 months, meet the implantation needs on the reconstruction sites.^24^ It is worth noting that there is often a higher exposure rate and failure rate in extensive bone augmentation.^50^ In Ciocca’s report, the exposure rate of titanium mesh was as high as 66%. However, the exposure of titanium mesh in these cases did not lead to significant impact on the results of bone augmentation, which may also be an advantage in the application of titanium mesh.^24^

### Application of titanium mesh in combination with other bone augmentation methods

In cases with a mass of alveolar bone defects or complex bone wall defect morphology, using onlay bone grafting or GBR alone may increase the risk of compensatory bone resorption or barrier membrane exposure.^51^ Hence, onlay bone grafting combined with GBR with titanium mesh is mostly used in this kind of cases. Under the premise of onlay bone grafting, deproteinized bovine bone mineral (DBBM) or particulate autologous bone was added around the autologous bone block to increase the volume of alveolar bone, reaching the required height and width of regular shape. Then, titanium mesh was used to stabilizing and protecting the graft material and autologous bone graft. The randomized controlled trial conducted by Roccuzzo et al. showed that onlay bone grafting combined GBR with titanium mesh for alveolar bone augmentation only achieved 13.5% bone resorption, while the bone resorption rate was up to 34.5% in cases using onlay bone grafting alone (*P* < 0.05).^52^ Also, the amount of autologous bone needed in onlay bone grafting is reduced. Titanium mesh bone augmentation can even be used to reconstruct alveolar bone defects after the failure of onlay autologous bone grafting. Zhou et al. covered hydroxyapatite particles with titanium mesh, reconstructed severely atrophied alveolar bone after the failure of onlay bone grafting, and still obtained an average of (4.2 ± 0.5) mm bone augmentation.^53^ After 3-year follow-up period, the bone around implant was still stable, proving that the bone augmentation of titanium mesh is effective and stable. Besides, GBR with titanium mesh can combined with tenting bone augmentation. Chan et al. conducted GBR with titanium mesh in combination with tenting screws for vertical alveolar ridge reconstruction, gained the average of (3.4 ± 1.9) mm bone height, while only Xenograft is used as graft material.^54^

## Surgical management of titanium mesh in bone augmentation

### Graft materials

Many studies focused on the bone graft materials covered by titanium mesh. Particulate grafts are ideal materials for filling different morphological bony defects, which can not only prevent the formation of interstitial space under the titanium mesh, but also have better osteogenic and osteoinductive properties compared to block bone grafts.^55^ The bone augmentation using particular autogenous bone grafts and titanium mesh has achieved good results in bone reconstruction and implant survival.^8^ However, for the bone quantity demand in the donor site, surgical cost, and patient’s condition restrict autogenous bone use, researchers are starting to look for ways to minimize the use of autogenous bones. Mixing autogenous bone with bone xenografts (such as inorganic bovine bone biomaterials) was proposed to reduce autogenous bone demand in bone augmentation. DBBM is one of the most used xenograft bone materials. Its high biocompatibility and low absorptivity enable it to associate with new bone, and maintain graft volume during the cancellous bone formation stage in bone reconstruction, as well as to increase bone density after bone formation.^10^ It has been used in many researches that applied xenograft alone or autogenous bone/xenograft bone mixture for bone augmentation.^10,34,38^ In recent years, clinical studies have used titanium mesh with different ratios of autogenous bone and xenograft (mostly DBBM) for bone augmentation; in clinical researches, the ratio of autogenous/xenograft bone is usually 50/50 or 30/70 (refs..^17,56–58^) Although the histological study demonstrated that when the ratio of autogenous bone in grafts mixture increases, the proportion of new bone tends to increase, there was no statistically significant difference to prove if autogenous bone plays an essential role in bone reconstruction.^54^ Research showed that after bone augmentation, DBBM particles are in close contact with the deposited new bone without inflammation sign in histology. And the histomorphological response with DBBM filling at the bone reconstruction area is the same as with autogenous bone filling.^59^ Thus, it can be considered that similar and predictable results can be obtained in both autogenous bone or different xenograft bone materials in bone reconstruction with titanium mesh.^59^

Lately, due to their ability to promote bone reconstruction and bone formation, osteoinductive factors based on recombinant human bone morphogenetic proteins (rhBMP) have become an alternative to traditional bone grafts. Among them, rhBMP-2 has been proved for extremely high osteoinductive potential, and is vital in the early stage of mesenchymal stem cell proliferating and differentiating into osteoblasts.^60^ Since rhBMP-2 is mostly stored in liquid form, absorbable collagen sponge (ACS) is often used as the carrier. And for lack of structural stability of the collagen carrier, titanium mesh is often used when using rhBMP-2/ACS for horizontal or vertical bone augmentation graft mateiral, to avoid the collapse of the osteogenesis space under pressure.^61^ Clinical research showed that compared to autogenous bone grafts, no significant differences in horizontal bone augmentation with rhBMP-2 were observed.^62^ Although the newly formed bone induced by rhBMP-2 material or autogenous bone grafts is histologically compatible with the residual alveolar bone, the induction of new bone mineralization leaded by BMP-2 will take a long time.^26,63^ Furthermore, the release process of rhBMP-2/ACS is uncontrollable, often released quickly after placement at the bone defect area, and may lead to postoperative edema.^25^

### Covering materials

The pores of titanium mesh are considered channels for connective tissue cells to form soft tissues under titanium mesh. Therefore, it is theoretically feasible to close the pores and avoid excessive soft tissue formation under titanium mesh by covering absorbable membrane upon titanium mesh. The biocompatibility of the absorbable collagen membrane can be utilized to promote mucosal healing. However, a retrospective cohort study found no statistical difference in the exposure rate of GBR with titanium mesh with/without covering absorbable collagen membrane. And even in cases that exposure did not occur, dense fibrous tissue was still found under the titanium mesh.^64^ Hence, it can be considered that covering an absorbable collagen membrane upon the titanium mesh cannot increase the mucosal healing speed, and it is also difficult to reduce excessive soft tissue formation under titanium mesh.

For another biomaterial commonly used in clinical, concentrate growth factors (CGF) was usually placed between titanium mesh and the gingival soft tissue. A clinical cohort study showed that patients treated with CGF during bone augmentation with titanium mesh healed better, and exhibited lower titanium mesh exposure.^65^ This phenomenon may be related to the promotion effect on angiogenesis and fibroblast differentiation of CGF, which can accelerate soft tissue healing, and protect titanium mesh and bone graft material under gingiva. Moreover, it can also reduce inflammation responses during wound healing.^66^

As the first generation of platelet concentrate, platelet-rich plasma (PRP) has a high platelet concentration and few natural fibrinogens. A randomized controlled trial revealed significant differences in complications and bone formation between the titanium mesh groups with or without PRP. And no titanium mesh exposure was found in the PRP group, while the mesh exposure rate was up to 28.5% in the control group. Moreover, though there is no significant difference, the average augmented bone amount of the PRP group ((3.5 ± 0.7) mm in vertical direction and (4.1 ± 0.6) mm in horizontal direction) was higher than that of the control group without PRP ((3.1 ± 0.8) mm in vertical direction and (3.7 ± 0.6) mm in horizontal direction) through the radiographic analysis.^65^ As the second generation of platelet concentrate and a fully autologous biomaterial, platelet-rich fibrin (PRF) is easier to prepare and apply than PRP.^67^ A retrospective study showed that in the bone augmentation surgery, the exposure rate with the group that solid advanced PRF (A-PRF) covering on the surface of titanium mesh (0.00%) is significantly less than that of the titanium mesh covered with the collagen membrane (23.5%; *P* < 0.05).^68^ This result may be related to the initial clot stabilization mediated by the fibrin in PRF on the one hand. On the other hand, PRF’s abilities of stimulating osteoblasts, periodontal ligament cells and epithelial cells, promoting bone defect repairing, wound healing, and micro-vascularization may also play an important role.^69–71^ Furthermore, based on PRF’s strong promoting ability in epithelialization and soft tissue healing, Ghanaati et al. tried not to close the wound after bone augmentation surgery with titanium mesh in a series of cases. A-PRF-loaded collagen matrix and A-PRF were used to cover titanium mesh and the gap between the flap margins, PTEF-based membranes or sterile latex was used to cover these materials for open healing. Though the titanium mesh was exposed over the healing stage, soft tissue still grew to close the wound without infection sign. Finally, desired bone augmentation effects and soft tissue esthetic results were obtained.^72^

### Fixation options of titanium mesh

In vitro study has found that the migration factor released by blood that in contact with the surface of titanium mesh plays a vital role in early cell recruitment. The interaction between blood and titanium mesh may regulate the early osteogenic microenvironment of bone healing and remodeling.^73^ Her et al. proposed a hypothesis that once the blood clot stabilizes in the bone augmentation area, the gingival connective tissue cells may be excluded like conventional GBR, and it is difficult for the soft tissue to move on the titanium mesh.^17^ Therefore, in GBR with titanium mesh, the mesh needs to be firmly fixed to prevent micromotion that affects the formation of blood clots.

According to the patient’s bone augmentation range and whether implants are implanted simultaneously, different methods can be used to fix the titanium mesh firmly. For titanium mesh bone augmentations with single tooth loss, in simultaneous implantation strategy, absorbable sutures can be used to fix titanium mesh, avoiding the impact of titanium screws placement on implants and additional incision for removing titanium mesh and screws in subsequent surgery, which reduces the patient’s trauma.^28^ For titanium mesh bone augmentations with single tooth loss in delayed implantation, the placement of titanium screws is not limited by the implant in two-stage implantation. Hence, titanium screws can be used to immobilize the titanium mesh, and the screws and titanium mesh can be removed at the subsequent implantation surgery.^26^ For patients with bone augmentation at multiple tooth sites, titanium screws can be fixed between multiple implants without affecting the implant during simultaneous implantation. And due to the relatively broad area of the bone defects that may cause movement on titanium mesh, titanium screws are usually used to fix the mesh firmly to facilitate blood clot stability and wound healing.^46^ Besides, for the titanium mesh preformed in the digital process, with the appropriate implant system, the corresponding accessories (height, cover cap, or healing abutment) can be directly connected to the implant to immobilize the titanium mesh. The cover cap or healing abutment can be respectively used for submerged and non-submerged implantation as appropriate.^11^

## Complications of titanium mesh, management, and prevention

For complications caused by GBR surgery with titanium mesh, surgical complications are generally less discussed, and researches pays more attention to the healing complications. During the healing stage, the wound dehiscence and consequent titanium mesh exposure are the main complications in GBR with titanium mesh. The stiffness of titanium mesh and its sharp edges generated in cutting and bending may cause detrimental stimulation on mucosal flaps, giving rise to the mucosal rupture and subsequent mesh exposure.^7^ The incidence of mesh exposure is mostly ~20%–30% (refs.,^17,52,65^) and the highest reported exposure rate is 66% (ref..^24^) According to exposure time, the exposure of titanium mesh can be divided into early exposure and late exposure. Exposure occurs within 4 weeks after bone augmentation is considered early exposure, which is likely to bring about detrimental effects. Specifically, it is manifested that an increase in fibrous tissue and a decrease in bone formation often occur at the bone defect area after early exposure. And it may damage the fusion of residual xenograft particles with the surrounding bone.^74^ Once early exposure is happened, the titanium mesh should be removed as soon as possible, and subsequent anti-infection operations should be conducted.^55^ The late exposure that occurred 4 weeks after bone augmentation may cause ~15%–25% of the graft resorption in the exposed area, making the exposed site’s bone volume slightly insufficient.^56^ After conducting anti-infection treatment and trimming sharp or irregular edges of exposed titanium mesh, the exposed area can heal without removing titanium mesh, which will not cause subsequent severe complications like infection and bone grafting failure, and will not influence bone regeneration in the defect area.^52^ No matter the early or late titanium mesh exposure occurred, it is not easy to cause consequent infection, which may be related to the smooth surface of titanium mesh, and the protective effect of the pseudo-periosteum formed between titanium mesh and the bone graft material.^37^ This layer of soft tissue protects the graft material from infection, and provides adequate blood supply, nutrition, and metabolic exchange, preventing the graft materials from absorption.^23^ Furthermore, researches indicated that for the periosteal-like tissue is still forming when the titanium mesh is early exposed, its role in preventing infection has not been exerted, which is one reason why early exposure needs special attention.^23,57^ In addition, except the time of exposure, the area of exposure also has an impact on bone formation. Complex bone augmentation procedure are more likely to cause large area of titanium mesh exposure, which may result in the less bone formation.^74,75^

In terms of strategies for preventing common complications during the application of titanium mesh, it is necessary to deal with preoperative preparations, surgical procedures, and postoperative care to control risk factors. In short, according to Fontana et al.’s summary of the complications during the use of nonabsorbable membranes in GBR technology,^76^ to prevent postoperative complications, the following should be done: before surgery, accurate evaluation of the patient’s general condition and local conditions of the operation area should be done, antibiotics should be taking preoperatively if necessary, and all sources of infection in the operation area and its vicinity need to be removed; during surgery, flaps should be designed to obtain adequate blood supply and facilitate flap closure, titanium mesh should be immobilize tightly, the buccal periosteal release and completely tension-free closure must be done, and deep mattress and single interrupted sutures are recommended; after surgery, systemic antibiotics and local disinfection care should be performed, and postoperative precautions should be explained to the patients clearly. Also, the application of custom-made titanium mesh through the digital process can prevent complications due to the avoidance of sharp edges caused by intraoperative bending.^77^

## Research progress of titanium mesh in bone augmentation

To avoid the occurrence of common complications, such as titanium mesh exposure after GBR with titanium mesh, a certain consensus in the clinical procedure has been reached. However, it is still far from enough. More and more researches focus on improving current titanium mesh bone augmentation procedure to reduce the complications and improve the bone augmentation effect. The research progress can be roughly divided into the progress in the digitalization and progress in the material modification of titanium mesh.

### Progress in digitization

Commercial titanium meshes usually have a plane shape with specified thickness and aperture. For local alveolar bone defects of different patients, the commercial titanium mesh needs to be manually bent and shaped into an appropriate shape during surgery to fit the alveolar ridge. This process often accompany with various inconveniences and disadvantages: bending intraoperatively is time-consuming, which dramatically increases the patient’s pain and the probability of infection;^78^ imprecise shape made by manual bending is hard to completely fit the alveolar ridge, making the outcome of bone augmentation become empirical; careless contact with gloves during the bending process may also lead to the degradation of biological properties on titanium mesh.^79^ In addition, the sharp edges on titanium mesh generated by the intraoperative bending are prone to cause mechanical stimulation on the mucosal flap, resulting in mucosal rupture and consequent exposure of the titanium mesh.^41^

**Fig. 2:**
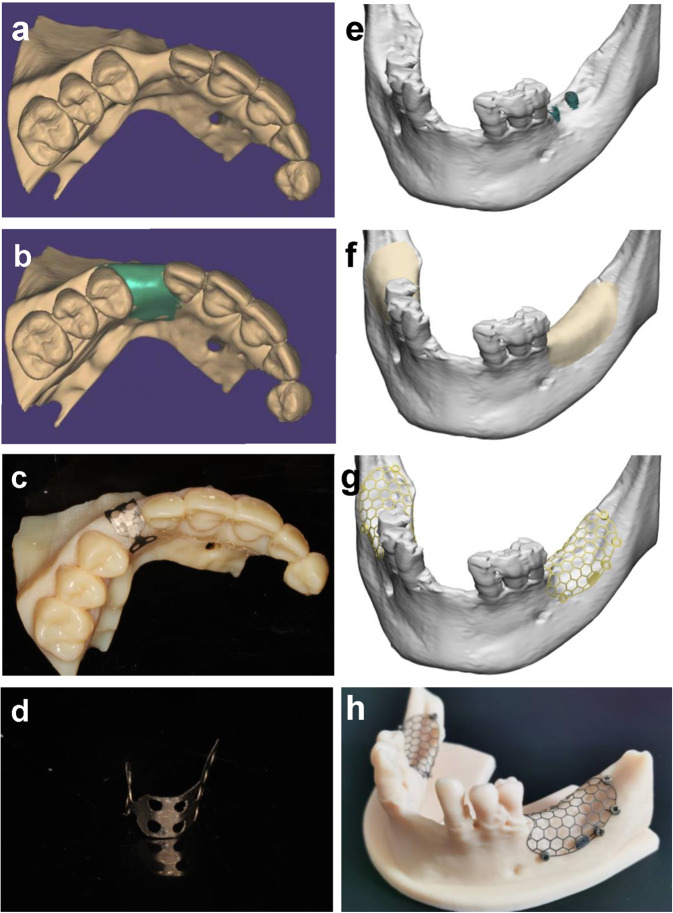
Digital procedure for preforming titanium mesh and three-dimensional-printed titanium mesh. **a**–**d** Digital procedure for preforming titanium mesh. **a** View of the three-dimensional virtual alveolar defect. **b** View of ideal augmented alveolar ridge after three-dimensional virtual bone augmentation according to arch form and expected implant position. **c** Titanium mesh was preformed on the virtual augmented maxilla model. **d** The preformed and trimmed titanium mesh. **e**–**h** Digital procedure for three-dimensional-printed titanium mesh. **e** Exposure of the implant was observed on the alveolar bone defect site after virtual implantation. **f** View of ideal augmented alveolar ridge after three-dimensional virtual bone augmentation according to arch form and expected implant position. **g** Three-dimensional design of the titanium mesh according to the ideal augmented ridge. **h** Three-dimensional-printed titanium mesh on the ideal augmented alveolar ridge model

Therefore, with the development of clinical requirements and digital implantation technology, custom-made titanium mesh has become one of the research trends in GBR with titanium mesh. Through a comprehensive preoperative assessment of the patient in cross-sectional imaging and digitalized research models, the ideal reconstructed alveolar ridge can be designed virtually according to arch form and expected implant position in computer-aided design (CAD) software.^80^ So far, two kinds of production methods of personalized titanium mesh have been developed: one of which is to directly design a matching custom-made titanium mesh on the planned reconstructed alveolar ridge model, and use CAD/computer-aided manufacturing (CAM) technology to print the titanium mesh through a 3D printing process.^68,81,82^ Another method is to preform the titanium mesh preoperatively on the 3D-printed planned reconstructed alveolar ridge model, and it can also form a personalized titanium mesh according to design requirements.^9,83,84^ Figure 2 shows the design and production process of these two kinds of digital titanium meshes. In contrast, with CAD/CAM technology, the unit structure, pore size, and thickness of 3D-printed titanium mesh can be adjusted according to demand; while the personalized titanium mesh made by preforming has lower technical sensitivity, and has better plasticity than 3D-printed titanium mesh.^24,85^

Compared to the conventional titanium mesh, the custom-made titanium mesh greatly shortens the operation time and is more suitable for the alveolar bone. The customized shape that entirely fits the alveolar ridge also reduces the retention need for using titanium screws.^38^ The rounded blunt edge formed by 3D printing reduces the risk of mucosal rupture and titanium mesh exposure, making it more accurate and less invasive.^11^ In 2015, Sumida et al.^77^ compared the personalized titanium mesh’s performance produced by a selective laser melting method with the conventional titanium mesh on bone augmentation in alveolar bone defects. This prospective controlled trial showed that, the time taken for bone augmentation surgery with the custom-made titanium mesh was significantly shorter than that of the conventional titanium mesh group. Moreover, the number of titanium screws used to immobilize is significantly fewer in custom-made titanium mesh groups than the conventional titanium mesh group. Although there was no significant difference in the titanium mesh exposure rate caused by wound dehiscence between the two groups, the personalized titanium mesh group (7.7%) was still less likely to cause titanium mesh exposure than that of the conventional titanium group (23.1%) in this study.^77^ Till now, many clinical studies showed that personalized titanium mesh plays an active role in shortening operation time, reducing the occurrence rate of bone augmentation complications, and improving the success rate of surgery.^10,82,86^

### Progress in material modification

#### Surface modification of titanium mesh

In recent years, the surface modification of titanium mesh has become a research trend to obtain better biological activity. Bioactive coatings for accelerating bone regeneration by improving the differentiation and proliferation of osteoblasts have been widely developed in tissue engineering. In a previous study by Nguyen et al., the effects of titanium mesh covered with calcium–phosphorus coating and untreated titanium mesh in GBR on the rat model were compared.^87^ It was found that compared to the untreated titanium mesh group, there is no soft tissue intervention under the titanium mesh of experimental group. And the bone density in experimental group is significantly higher (*P* < 0.05), which improves the structural durability of bone regeneration. In his latest research, strontium is used to promote osteoblasts’ proliferation and differentiation, and inhibit osteoclasts’ activity. It was combined with a calcium–phosphorus coating on the surface of titanium mesh, to test its role in GBR on the rat model.^88^ Compared to untreated and calcium–phosphorus-coated titanium mesh, the strontium–calcium–phosphorus coating is more uniform and denser, enabling higher bone density in bone regenerated area, improving the osseointegration between bone and titanium mesh, and preventing infiltration of soft tissue in early stage of healing. This study believes that proper coating treatment on the titanium mesh can form stable new bone without specific adverse reactions and shorten the time taken for GBR.

#### Photofunctionalization of titanium

Studies have shown that the titanium mesh surface is covered with hydrocarbon molecules, which will gradually accumulate over time, causing the loss of titanium’s hydrophilicity, and damaging the bone conduction and osseointegration ability of titanium. This process is called titanium biological aging.^89^ However, the ultraviolet treatment can change the surface properties of aged titanium: during the ultraviolet treatment, the hydrocarbon pollution on the surface of titanium can be removed. Meanwhile, the titanium mesh has obtained super-hydrophilic, and its surface charge is changed from negative to positive.^90^ This ultraviolet treatment is defined as the titanium’s photofunctionalization, which can enhance the biologic capabilities and stability of titanium mesh, increase the adhesion and retention of osteoblasts on the titanium mesh, and it is considered as a method to solve the biological aging problem. Animal experiment research showed that the photofunctionalized titanium mesh attracts more blood from the bone defect area than the untreated titanium mesh dose. The super-hydrophilic surface is deemed more conducive to the migration of osteoblasts in the bone regeneration area.^89^ It can be considered that photofunctionalization is of positive significance in GBR with titanium mesh. Also, similar to titanium implants, inadvertent contact between titanium mesh and the glove during surgery will also cause biological contamination of titanium mesh, resulting in a decrease in the biological activity of titanium mesh. For the pollution caused by glove contact, although photofunctionalization cannot wholly remove the impurity particles on titanium, it can remove carbon and some other impurities, restore the biological activity of titanium mesh, and enhance its surface adhesion to osteoblasts.^79^ Due to the simple procedure of photofunctionalization technology to treat titanium surface, and the consistently positive results in clinical studies of photofunctionalized titanium implants, clinical trials of photofunctionalized titanium mesh for bone augmentation are expected carrying out soon.

## Conclusion

For the reconstruction of alveolar ridge defect, GBR technology is convenient and predictable. Due to its broad application indications, suitable elasticity and plasticity, and excellent mechanical properties, titanium mesh can meet bone augmentation needs in most clinical situations, making it stand out from traditional GBR barrier membranes. With the research progresses, although there are still problems, such as high exposure rate in its application, the clinical procedures of GBR with titanium mesh are continuously improved to shorten the surgery time, reduce patient trauma, and increase the success rate of bone augmentation. In conclusion, the titanium mesh applications in GBR have achieved feasibility and predictability in horizontal, vertical, and three-dimensional bone augmentation. It is an excellent bone augmentation method for alveolar ridge reconstruction.
